# B Cell Metabolism and Autophagy in Autoimmunity

**DOI:** 10.3389/fimmu.2021.681105

**Published:** 2021-06-07

**Authors:** Iwan G. A. Raza, Alexander J. Clarke

**Affiliations:** ^1^ Medical Sciences Division, University of Oxford, Oxford, United Kingdom; ^2^ Kennedy Institute of Rheumatology, University of Oxford, Oxford, United Kingdom

**Keywords:** autophagy, metabolism, autoimmunity, B cell, SLE - systemic lupus erythematosus, B cell development and differentiation

## Abstract

B cells are central to the pathogenesis of multiple autoimmune diseases, through antigen presentation, cytokine secretion, and the production of autoantibodies. During development and differentiation, B cells undergo drastic changes in their physiology. It is emerging that these are accompanied by equally significant shifts in metabolic phenotype, which may themselves also drive and enforce the functional properties of the cell. The dysfunction of B cells during autoimmunity is characterised by the breaching of tolerogenic checkpoints, and there is developing evidence that the metabolic state of B cells may contribute to this. Determining the metabolic phenotype of B cells in autoimmunity is an area of active study, and is important because intervention by metabolism-altering therapeutic approaches may represent an attractive treatment target.

## Introduction

B lymphocytes play a crucial role in immune responses against pathogens and tumours through the production of protective antibodies. They are also implicated in the development and control of autoimmunity, in which the immune response is directed towards self-antigens. Various B cell populations display the capacity for both protective and self-destructive activity. The best understood B lymphocytes are conventional bone marrow-derived B2 cells, which mediate adaptive humoral responses. In various autoimmune diseases, including rheumatoid arthritis (RA) and systemic lupus erythematosus (SLE), B2 cells are responsible for autoantibody production, the presentation of endogenous peptides to self-reactive T cells, and the secretion of proinflammatory cytokines (1–5). Additional to B2 cells are a distinct population of innate–like antibody–secreting lymphocytes, known as B1 cells. Generated in the foetal liver, B1 cells are found with the peritoneum and are capable of resting self–renewal. B1 cells produce natural IgM against bacterial polysaccharide antigens and exhibit a significant degree of self–reactivity (6, 7). The autoantibodies and cytokines produced by B1 cells can mediate autoimmunity (8, 9). However, B1 cell–derived autoantibodies also facilitate the clearance of apoptotic cells, a major source of autoantigens, and appear to promote intestinal homeostasis (10–12). Meanwhile, regulatory B cells (B_regs_) play a well–defined role in tolerogenesis, secreting immunoregulatory cytokines such as IL–10 and TGF–β, and enacting contact–dependent suppression of self–reactive lymphocytes (13).

The causes of B cell dysfunction in autoimmunity remain incompletely defined. Metabolism, although a well–established regulator of cellular activity, has only relatively recently been appreciated as a determinant of B cell function in health and disease. Physiologically, metabolism enables proper B cell development, differentiation and antibody secretion (14, 15). Unsurprisingly, the contrasting phenotypes of B cells at different stages of development and maturity are reflected in significant variations in metabolic activity (16, 17). Moreover, different B cell subsets, particularly B1 and B2 cells, utilise divergent metabolic pathways (18). Metabolism forms an important component of deletional and anergic checkpoints against autoimmunity, and metabolic dysregulation is associated with the escape from tolerogenic checkpoints and the enhanced functionality of self–reactive B cells (19–22). As well as traditional aspects of cellular metabolism, B cells depend on autophagy, a mechanism of degradative processing, principally of damaged cellular components. Autophagy maintains metabolic homeostasis during nutrient deprivation and supports long–term plasma cell (PC) viability (23). Pathologically, autophagy appears to support self–reactive B cells in subverting autoimmune checkpoints, to undergo activation by innate immune signals, and present autoantigens to T lymphocytes (24–26).

In this review, we describe B cell metabolism and autophagy in health, highlighting their roles in immune homeostasis. We then discuss the metabolic and autophagic abnormalities seen in autoimmune B cells, and how these may promote self–destructive responses. Finally, the potential of B cell metabolism and autophagy as therapeutic targets in autoimmunity will be explored. This review focuses primarily on conventional B2 cells, which are relatively well defined metabolically, although comparisons are made to B1 cells and B_regs_ where possible.

## Metabolism and Autophagy in B Cell Development

The development of conventional B2 B cells must generate a vast and hugely diverse repertoire of cells capable of antibody secretion, whilst purging cells with self–reactive antigen specificities. Moreover, rapid growth and proliferation must be achieved in the metabolically challenging environment of the bone marrow, requiring the careful balancing of anabolic and catabolic signalling. The former is largely mediated by c–Myc and mitochondrial target of rapamycin complex (mTORC) signalling, which fuel protein synthesis and cell growth by upregulating glycolysis and oxidative phosphorylation (OXPHOS) (14, 27, 28). mTORC signalling plays a crucial role in successful B cell development (27, 28). In mice, deletion of the mTORC1–associated protein Raptor prevents the interleukin (IL)–7–driven development of pro–B cells (14, 29). Without mTORC1 signalling, pro–B cells are less able to transition into pre–B cells, the precursors in which the pre–B cell receptor (BCR), consisting of mature immunoglobulin heavy chains and surrogate light chains, is expressed (14, 29). Unlike mTORC1, the role of mTORC2 in early B cell development has been contested, although it appears to facilitate peripheral B cell maturation (14, 30). Specifically, mTORC2 has been implicated in regulating mTORC1 and c–Myc activity during the terminal stages of B cell development (31).

While mTORC signalling is important during B cell development, excessive anabolic activity is detrimental. Indeed, B cell development is compromised at the large pre–B cell stage following the deletion of Fnip1 (32). Fnip1 interacts with 5’ adenosine monophosphate–activated protein kinase (AMPK), an energy stress sensor and catabolic regulator which opposes mTORC1. Although AMPK can promote catabolism in the absence of Fnip1, its ability to inhibit mTORC signalling is impaired (32). The metabolic stress resulting from unrestrained anabolism renders pre–B cells more vulnerable to apoptosis following pre–BCR cross–linking (32). The concept of metabolism as a regulator of cell death, controlling B cell precursor viability in response to antigen stimulation, has implications for tolerogenic checkpoints against autoimmunity (33).

The large pre–B cell stage, during which the pre–BCR is expressed on the cell surface and tested for affinity towards self–antigens in the bone marrow, represents both an autoimmune checkpoint and a period of metabolic vulnerability (34). Although its necessity during B cell development is controversial, glucose metabolism represents one example of this vulnerability (20, 32, 35). The rapid proliferation of large pre–B cells is believed to be sustained through upregulated glucose metabolism, given that large pre–B cells import more glucose than other precursor populations (16, 35). Large pre–B cells experience significant oxidative stress and are vulnerable to glycolytic inhibition, which impairs their transition into small pre–B cells (16, 35). Signalling through an autoreactive pre–BCR drives hyperactivation of the phosphoinositide 3–kinase (PI3K)–protein kinase B (Akt)–mTORC1 pathway, with the resulting metabolic stress inducing negative selection (19). Activation of a non–autoreactive pre–BCR does not affect Akt activation or viability in pre–B or leukemic pre–B (pre–B ALL) cells (19, 36). In contrast, activation of an autoreactive pre–BCR induces rapid, Akt–dependent cell death (19). In pre–B ALL cells, deletion of the PI3K inhibitor phosphatase and tensin homologue (PTEN) increases glycolytic flux, although elevated anabolism results in ATP depletion and cell death (19). These changes are reversed by the mTORC1 inhibitor rapamycin, suggesting that hyperactivation of the PI3K–Akt–mTORC1 pathway downstream of an autoreactive pre–BCR results in an energy crisis (19). PTEN plays a vital role in the development of pro–B cells, reducing their susceptibility to apoptosis (14). While PTEN deletion affects non–metabolic features of B cell precursors, such as B lymphoid transcription factor expression, these results further highlight the importance of balanced metabolic programmes during B cell development (14).

Alongside controlling other aspects of development, B lymphoid transcriptional factors themselves impose metabolic restriction upon B cell precursors, perhaps to enable hyperactivation–induced cell death (37). Mutations in the transcription factors *PAX5* and *IKZF1* are commonly seen in acute lymphoblastic leukaemia, suggesting that their expression may confer a selective disadvantage (37). The inducible reconstitution of *PAX5* and *IKZF1* in pre–B ALL cells reduces glucose uptake and ATP synthesis, promoting cell death (37). Notably, the B cell–specific expression of a non–functional *IKZF1* predisposes mice to the development of autoimmunity, supporting the idea that these transcription factors may play a tolerogenic role during B cell development (38). Metabolic restriction is also a prominent feature of B cell anergy. Along with apoptosis and receptor editing, tolerogenesis can be exerted on self–reactive B cells and their precursors through the induction of anergy, rendering B cells hyporesponsive to antigenic stimulation. Anergy is a major mechanism of tolerising early transitional B cells following egress from the bone marrow. Anergic B cells are characterised by suppressed PI3K signalling and impaired metabolic reprogramming in response to BCR or Toll–like receptor (TLR) 4 stimulation (20, 39). Presumably, metabolic suppression increases the activation threshold of anergic self–reactive B cells.

**Figure 1:**
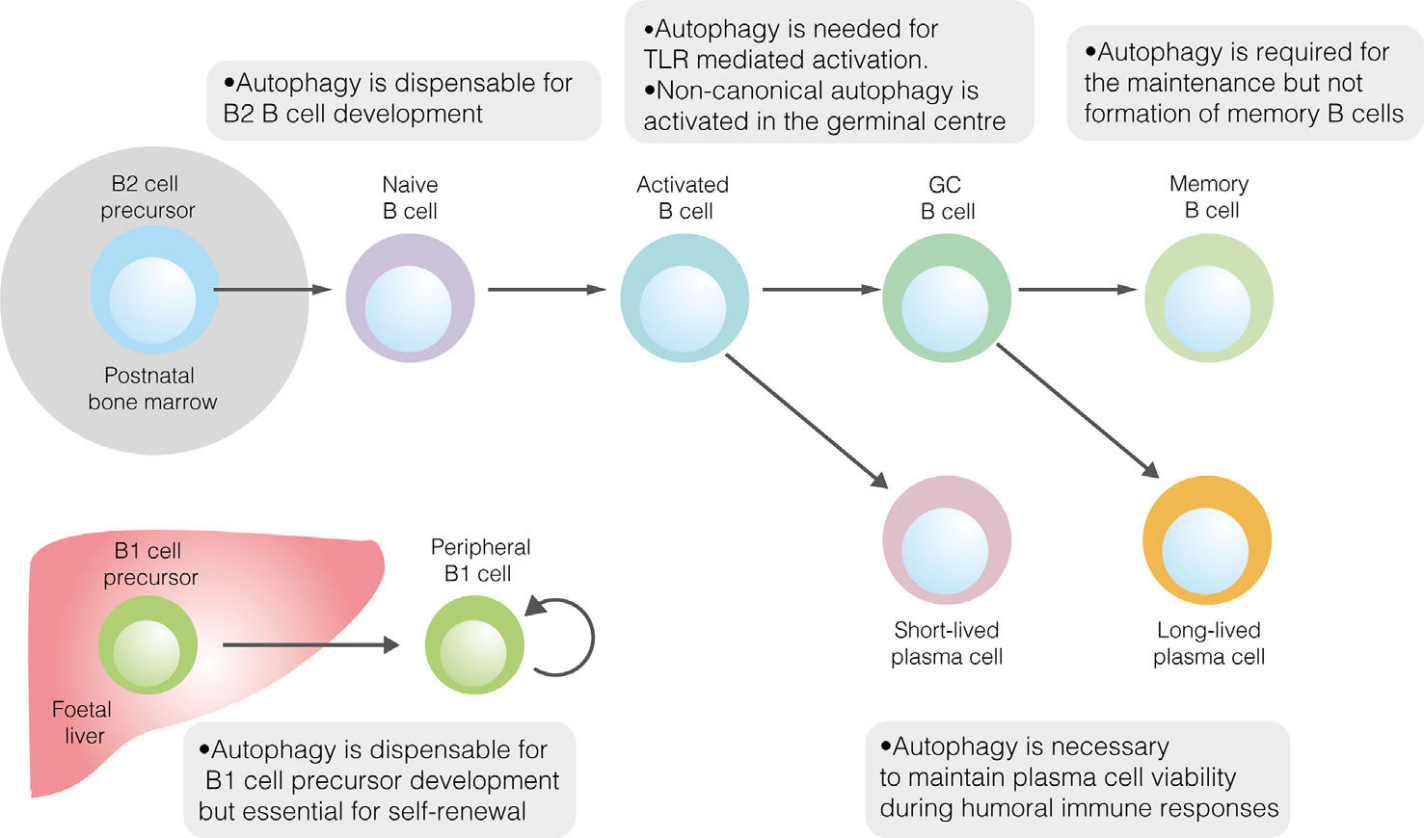
The role of autophagy in developmental and mature B cells. Autophagy performs homeostatic roles in various B cell subsets. It is dispensable for the development of both B1 and B2 cells but is required for the peripheral self–renewal of B1 cells. Regarding B2 cells, autophagy is necessary for TLR–mediated, but not antigen–driven, activation. Autophagy also plays important roles in sustaining plasma cell immunoglobulin production and ensuring memory B cell survival.

Given its role in promoting metabolic homeostasis, the role of autophagy in B2 cell development has been explored (**Figure 1**) (40). Reconstitution of the foetal livers of *Rag1^–/–^*mice with cells lacking the key autophagy gene *Atg5* demonstrates a developmental block at the pre–B cell stage (41). When *Atg5* deletion was restricted to mature B cells, splenic and lymph node B cell populations were unaffected, implying that autophagy is necessary for the development, but not peripheral maintenance, of B2 cells (41). However, pro– to pre–B cell transition occurs in the absence of *Atg5* expression following conditional deletion using Cd79a–cre (40). In contrast, autophagy was necessary to maintain populations of mature B cells in the periphery (40). That autophagy may be dispensable in B2 cell precursors is perhaps unsurprising, given the crucial developmental role of the autophagy inhibitor mTORC1 (14).

In contrast to B2 cell development in bone marrow, innate–like B1 cells develop in the foetal liver before migrating principally to the peritoneum and pleura (42). Given that B1 cells occupy different niches and rely upon self–renewal for population maintenance, their metabolic phenotype unsurprisingly differs from that of conventional B2 cells (42). Compared to follicular B2 cells, peritoneal B1 cells are characterised by greater glucose uptake, higher rates of glycolysis and OXPHOS and heightened sensitivity to glycolytic inhibition (18). Mechanistically, elevated glucose metabolism is likely driven by high levels of c–Myc expression (18, 43). In keeping with their localisation in the lipid–rich environment of the peritoneum, B1a cells extensively acquire exogenous fatty acids, while also utilising endogenous fatty acid synthesis (18).

Autophagy has also been explored in B1 cells (**Figure 1**). The loss of autophagic flux in mature B cells leads to significant depletion of B1a, though not B1b, cells (41). The specific role of autophagy in B1 cell development has only recently been explored (18). In mice, the B1 progenitor population is unaffected by the absence of autophagy, suggesting that autophagy is not required for B1 cell development (18). This conclusion is supported by temporal changes in the murine B1a cell population in the absence of autophagy. While the B1a cell compartment is normal at two weeks of age, by 12 weeks it is dramatically smaller than in wildtype mice (18). Together, these results suggest that autophagy is needed for peripheral self–renewal but not the differentiation of B1a cells. In peritoneal B1a cells, autophagy appears to be important for controlling the expression of metabolic genes, fatty acid uptake and degradation of lipid droplets (lipophagy) (18).

## B Cell Metabolism Following Activation

In the periphery, naïve B cells are maintained in a state of metabolic quiescence, which likely promotes their long–term viability (44). The maturation of transitional B cells, which egress from the bone marrow and are peripherally tolerised, to follicular cells, is associated with a reduction in OXPHOS, glycolysis and protein synthesis (44). An increased ratio of AMPK: mTORC1 activity suppresses anabolism and exposure to oxidative stress (44). However, the survival of naïve B cells requires some degree of metabolic activity. Their maintenance depends on tonic signalling *via* the BCR and B cell activating factor (BAFF) receptor, both of which activate PI3K (45, 46). Notably, BAFF signalling upregulates the expression of enzymes involved in glucose metabolism (46). IL–4, another extrinsic signal required by naïve B cells, upregulates glucose uptake and glycolysis in murine B cells in a PI3K–independent manner (47). Although these signals promote glucose metabolism, naïve B cells appear to rely heavily on fatty acid oxidation to generate ATP (20).

During B cell activation, this relatively inactive metabolic state is rapidly reversed, enabling growth and proliferation. Stimulation of murine B cells *via* their BCR increases mitochondrial mass and PI3K–dependent glucose uptake (20, 48). In mice, elevated glucose import is enabled by Glut1 upregulation, although human B cells express relatively little Glut1 and may rely on other transporters (15, 48). However, the nature of glucose metabolism in activated B cells has been contested. Activation *via* TLR4 or the BCR had been thought to upregulate both glycolysis and OXPHOS in a balanced manner, with only the former considered essential for successful antibody responses (20, 48, 49). A recent analysis, combining RNA sequencing and stable isotope tracing, has instead suggested that mitochondrial metabolism, but not glycolysis, is upregulated during B cell activation (17). While glucose did flux through glycolysis, much was diverted towards the pentose phosphate pathway, providing substrates for nucleotide synthesis and the management of oxidative stress (17, 48). While excessive oxidative stress compromises cell viability, reactive oxygen species (ROS) represent important signalling molecules in B cells. ROS levels, determined in part by mTORC1 activity, provide instructive signals during differentiation through the regulation of haem synthesis (50, 51). As well as feeding the pentose phosphate pathway, imported glucose is used for *de novo* lipogenesis (17, 52). Fatty acid oxidation, which is extensively utilised by naïve B cells, is downregulated following activation (20). Instead, glutamine appears to represent a major substrate for mitochondrial respiration following B cell activation (17). In activated B cells, mitochondrial respiratory capacity and homeostasis are maintained by AMPK (53).

Successful antigen–driven B cell activation requires T cell costimulation, creating a checkpoint against autoimmunity. T cells are subject to stringent tolerogenesis and play an important role in preventing the activation of self–reactive B cells which have escaped other tolerance mechanisms. Metabolism contributes to the function of this post–activation checkpoint (54). In murine B cells, metabolic activation following anti–IgM stimulation is not sufficiently supported by increased mitochondrial biogenesis or glucose uptake (54). This results in ROS accumulation and apoptosis driven by mitochondrial dysfunction, which is averted by the provision of T cell help (54). Overall, antigen engagement appears to create a brief window within which B cells must obtain costimulation to avoid activation–induced cell death.

Following B cell activation, antibody–secreting cells are generated through PC differentiation. The vast quantity of immunoglobulin secreted by PCs necessitates an increase in protein production capacity. During PC differentiation, the transcription factors Blimp1 and Xbp1 mediate substantial expansion of the endoplasmic reticulum (ER) (55). However, high levels of immunoglobulin production result in the accumulation of misfolded proteins and ER stress. To maintaining metabolic homeostasis, PCs utilise both antioxidant responses and the unfolded protein response (UPR), which limits mRNA translation while enhancing protein folding capacity and the ability of the ER to degrade misfolded proteins (56, 57). While Xbp1 and Blimp1 control the UPR in PCs, it has recently been shown that mTORC1 mediates a predictive UPR, which precedes antibody secretion (58, 59). Misfolded proteins are also degraded *via* the proteasome. Surprisingly, proteasome capacity decreases progressively during PC differentiation in mice, with proteasome inhibition reducing PC viability (60). Excessive ER stress has been suggested as a factor limiting the lifespan of short–lived PCs (SLPCs), although the expression ER stress response genes is equivalent in SLPCs and long–lived PCs (LLPCs) (61, 62).

PCs require a high level of metabolic activity to fuel extensive immunoglobulin production. Metabolic remodelling occurs during PC differentiation, with Blimp1 promoting oxidative metabolism (49). Basal OXPHOS is fed by long–chain fatty acids, with glucose–derived pyruvate acting as a biosynthetic substrate and providing spare respiratory capacity in LLPCs (15). The majority of glucose taken up by PCs is diverted towards antibody glycosylation *via* the hexosamine pathway, although glycolysis can supplement ATP production (15, 49). Predictably, PCs utilise amino acid metabolism: the expression of CD98, a component of many amino acid transporters, is induced by Blimp1, and is upregulated in LLPCs compared to SLPCs (58, 61). Amino acids are used during antibody synthesis, glutamine is used to generate several of these amino acids, while also acting as a substrate for oxidative metabolism (61). The activation of mTORC1, which is crucial for PC differentiation and optimising antibody output, is supported by high levels of amino acids (58, 63). In mice, AMPK is dispensable for LLPC persistence but restrains antibody synthesis, promoting metabolic homeostasis (53). As in other biological processes, a delicate balance of mTORC1 and AMPK signalling appear to provide an optimal metabolic environment for PC function.

**Figure 2:**
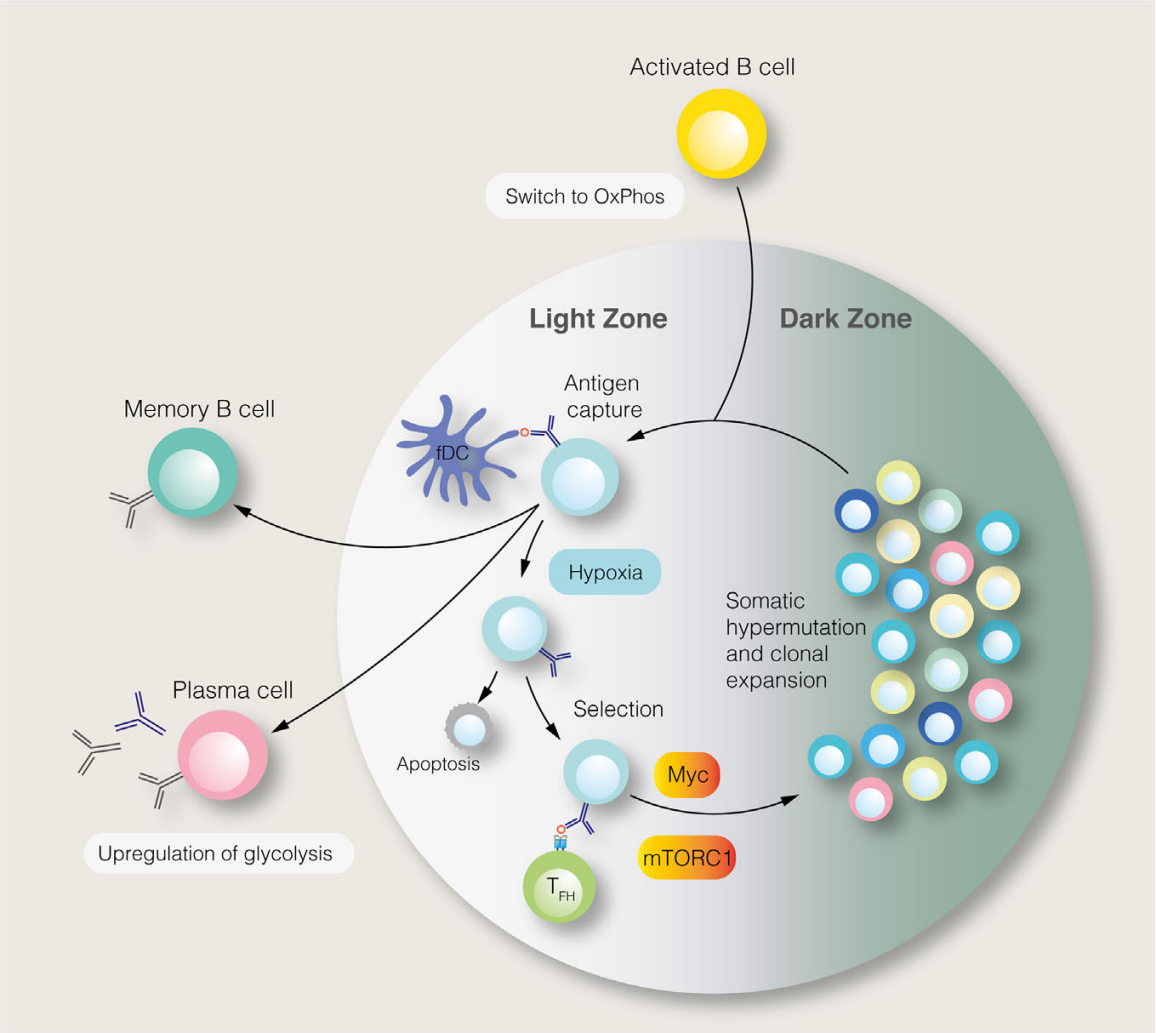
The germinal centre reaction and its regulation by metabolic signals. In the germinal centre light zone, centrocytes capture antigens from follicular dendritic cells (fDC). Centrocytes with the highest antigen affinities are most likely to acquire antigens and present antigen–derived peptides to follicular helper T (T_FH_) cells. Centrocytes which receive pro–survival signals from T_FH_ cells may migrate to the dark zone, where, as centroblasts (CB), they undergo somatic hypermutation and clonal expansion. This process generates B cells with new antigen specificities. The proliferation of centroblasts is enabled by mTORC1 and c–Myc signalling in the light zone, although this anabolic phenotype is restrained by germinal centre hypoxia. Instead of entering the dark zone, centrocytes, may differentiate into long–lived plasma cells and memory B cells.

SLPCs are generated following initial B cell activation, while LLPCs, characterised by affinity maturation and class–switch recombination, are produced within germinal centres (GC) of secondary lymphoid organs (**Figure 2**). Each GC consists of light and dark zones, defined by histological appearance. Within the light zone, B cells of different antigen specificities compete for pro–survival signals provided by follicular helper T (T_FH_) cells. B cells with high antigen affinities are more likely to receive sufficient T cell help, enabling them to migrate to the dark zone, where they undergo clonal expansion and somatic hypermutation. To fuel this proliferation, light zone B cells, known as centrocytes, adopt an anabolic phenotype mediated by mTORC1 (64). Centrocytes which receive CD40–mediated signals, representing T_FH_ cell help, activate mTORC1 to promote glucose uptake, cell growth and ribosomal biogenesis (64). mTORC1 activation, specifically in the light zone, is essential for GC function (64). The activity of centrocytes also requires PI3K signalling (65). Upstream of the PI3K–Akt–mTORC1 pathway, the GTPase R–Ras2 couples T_FH_–derived signals to mitochondrial and glycolytic metabolism (66). Recently, however, it has been shown that GC B cells rely on fatty acid oxidation for ATP production, utilising glycolysis minimally (67). Despite being highly active, GCs are characterised by nutrient deprivation and hypoxia, particularly within the light zone (68, 69). Within the GC, mTORC1 signalling is opposed by HIF–1α and glycogen synthase kinase 3, which promotes B cell viability during nutrient deprivation (68, 69). Interestingly, B cell metabolism changes as the GC reaction progresses (70). Over time, the GC goes from producing predominantly memory cells to LLPCs, with this switch thought to be partly mediated by metabolic changes (70). Indeed, a subset of B cells in the GC, characterised by reduced mTORC1 and c–Myc signalling, display a propensity for memory B cell differentiation (71).

Little is known about the metabolism of memory B cells themselves, although they are thought to be relatively quiescent metabolically. Unlike naïve B cells and PCs, memory cells are capable of BAFF–independent survival (72). AMPK appears to play a crucial role in maintaining metabolic homeostasis in memory B cells, supporting mitochondrial function and preventing excessive oxidative stress (53). Memory B cell inactivity is rapidly reversed upon antigen re–exposure, enabling PC differentiation. During secondary humoral immune responses, the balance between mTORC1 and AMPK signalling controls the nature of this differentiation (22, 53).

As with memory B cells, the metabolic phenotype of unconventional B cell subsets is largely unexplored. Recently, the production of the immunoregulatory cytokine IL–10 by B_regs_ has been shown to depend on both cholesterol metabolism and HIF–1α (73, 74). However, more work is needed to metabolically characterise B_regs_, as well as B1 cells.

## Autophagy in Activated B Cells

As with other aspects of metabolism, autophagy plays a crucial role in B cell function post–activation. In the initial stages of B cell activation, the differential regulation of autophagy by BCR engagement and T cell costimulation has been proposed to create an autoimmune checkpoint (75). While BCR stimulation of primary B cells promotes autophagy and triggers apoptosis, concomitant stimulation *via* the CD40 coreceptor induces a more modest rise in autophagy levels, limiting cell death (75). Although the amelioration of B cell autophagy by CD40 engagement has been disputed, the non–optimal induction of autophagy in the absence of costimulation may sensitise self–reactive B cells to cell death pathways (75, 76). Acutely, increased autophagic flux following BCR engagement is driven by the upregulation of non–canonical autophagy, which is resistant to inhibition by bafilomycin A1 (76). In contrast, bafilomycin–sensitive canonical autophagy is temporarily inhibited following BCR activation, likely due to increased mTORC1 activity (76).

While autophagy is induced by BCR signalling, it appears to be dispensable for BCR–driven B cell activation, given that proliferation following anti–IgM stimulation is unaffected in the absence of autophagy (**Figure 1**) (40). B cell autophagy is also induced by stimulation with TLR ligands, although in this context it appears to be necessary for plasmablast differentiation and survival (**Figure 1**) (23, 76). It was initially demonstrated that plasmablast differentiation did occur in autophagy–deficient B cells following stimulation with the TLR4 ligand lipopolysaccharide (LPS), although significant cell death was incurred (23). Furthermore, cells in which *Atg5* deletion was not penetrant were selectively enriched during differentiation (23). It has been subsequently reported that stimulation with LPS or the TLR9 ligand CpG leads to impaired plasmablast differentiation and viability among B cells lacking the autophagy genes *Atg5* or *Atg7*, characterised by the accumulation of damaged mitochondria and reduced expression of B cell transcriptional regulators (25, 40, 77). The effect of autophagy on plasmablast differentiation likely varies between studies according to the extent of metabolic stress induced during different protocols.

In the context of T cell–dependent (TD) antigens, costimulation requires antigen presentation on class II major histocompatibility complexes (MHC–II) to cognate T cells. Several early studies demonstrated that the ability of B cells to present antigens on MHC–II was altered following pharmacological inhibition of autophagy or its induction through starvation (78–80). Of note, these studies were conducted with Epstein–Barr virus (EBV)–infected B cells. EBV infection modifies B cell antigen presentation, limiting the physiological relevance of this model (24). More recently, autophagy has been implicated in the polarisation of internalised antigens following BCR engagement (81). In the absence of autophagy, the colocalisation of antigen–BCR complexes with MHC–II–containing vesicles is disrupted, particularly in the case of particulate antigens (81). While partial relocalisation of the BCR–antigen complex is sufficient for B cells to present peptides from soluble antigens on MHC–II, the presentation of particulate antigen epitopes to cognate T cells is compromised (81). Overall, autophagy appears to be necessary for the presentation of some, but not all, antigens by B cells.

The GC reaction, which enables the generation of memory B cells and LLPCs, is also characterised by extensive B cell autophagy. Of note, levels of canonical autophagy are relatively low, likely due to the upregulation of mTORC1 by T_FH_ cell–derived costimulation (76). The high overall levels of autophagy in GC B cells are skewed towards non–canonical flux, regulated by WIPI2 (76). The role of autophagy in GC function has been questioned, as neither its macroscopic appearance nor the extent of B cell affinity maturation is affected by B–cell–specific *Atg5* deletion (**Figure 1**) (23, 82). However, while it has only been shown that class III PI3K–independent non–canonical autophagy occurs in the GC, some forms of non–canonical autophagy proceed in the absence of *Atg5* itself (76, 83). It is possible that non–canonical autophagy occurs in the GC, and in other B cell subsets, following the deletion of core autophagy genes.

Given its roles in metabolic homeostasis and the degradation of misfolded proteins, it is unsurprising that autophagy appears to play an important role in the maintenance of antibody responses in both SLPCs and LLPCs (**Figure 1**). However, the precise effects of its inhibition are contested. As discussed in the context of plasmablast differentiation, there is disagreement as to the necessity of autophagy for initial antibody production following LPS–induced activation. *Atg7* deletion has been shown to compromise short–term IgM production, in line with failed plasmablast differentiation (25). However, it has also been reported that B cells lacking *Atg5* display elevated short–term antibody production following LPS stimulation, although in this study plasmablasts were characterised by ATP deficiency and elevated ER stress (23). This discrepancy, as in differences seen in plasmablast differentiation, may be caused by differences in the degree and timing of metabolic stress experienced by activated B cells. The idea that autophagy may restrain short–term immunoglobulin output suggests that it instead promotes long–term PC viability (23). In support of this conclusion, IgM and IgG responses to T cell independent (TI) pneumococcal polysaccharide antigens and the TD antigen NP–CGG were diminished in B cell autophagy–deficient mice, as was long–term LLPC survival (23). In contrast, humoral immunity against the TI hapten NP–Ficoll was unaffected by the loss of B cell autophagy in mice, perhaps due to continual B cell activation by this antigen (23). At the same time, another report found that early antibody responses to hapten–conjugated TD and TI antigens, as well as helminths, were diminished in B–autophagy–deficient mice (77). Subsequently, B cell autophagy was suggested to be important to antigen–specific IgM but not IgG responses against the TD antigen ovalbumin in mice (40). Autophagy was concluded to be important for PC survival, with LLPCs displaying greater resistance to a short–term loss of autophagy than SLPCs (40). Meanwhile, a separate study found that the murine humoral response to the TD antigen NP–KLH was largely unaffected by the loss of B cell autophagy, although anti–NP IgG1 trended towards a decrease six weeks post–immunisation (84). Clearly, there is significant heterogeneity in results, likely arising from differences in immunisation protocols. However, these results together suggest an important role for autophagy in maintaining PC viability over time.

To enable their longevity, LLPCs would logically utilise autophagy to a greater degree than SLPCs. Accordingly, the extent to which PCs utilise autophagy varies according to cellular lifespan. Among adult human bone marrow PCs, the expression of autophagy–associated genes and the prevalence of autophagic LC3B–II punctae are greater among LLPCs than SLPCs (85). In agreement with human data, autophagosomes are more prevalent in murine PCs with longer half–lives (61). Autophagy also appears to support the long–term survival of murine memory B cells, thus promoting the maintenance of immunological memory (**Figure 1**) (82, 84). Expression of autophagy–associated genes is higher in memory B cells than other B cell subsets, suggesting an important role in their function (84). In B cell autophagy–deficient mice, secondary humoral immunity against NP–KLH is affected to a much greater degree than the primary response, indicating a failure of B cell memory (84). While the number of memory cells formed two weeks post–immunisation was normal in mice lacking B cell autophagy, their number was markedly diminished by eight weeks, indicating that autophagy is not necessary for memory B cell formation, but is needed to maintain this population (82). Autophagy–deficient early, but not late, memory B cells are able to mount functional immune responses following antigen re–exposure (82). The survival of autophagy–deficient memory B cell survival is partially restored by NecroX–2, a necrosis inhibitor which reduces oxidative stress (84). Recently, it has been shown that mitochondrial autophagy, which helps to limit oxidative stress, is regulated in memory B cells by AMPK (53). Together, these results implicate autophagy as an important pro–survival mechanism in memory B cells, as well as PCs.

## B Cell Metabolism in Autoimmunity

The past decade has seen an explosion in research exploring the association between immunometabolism and autoimmunity (86). Within this field, B cells have received relatively little attention. Nevertheless, there are clear indications that the metabolic profile of B cells is disturbed in autoimmunity, particularly in SLE. Importantly, dysregulated B cell metabolism has been implicated in promoting and exacerbating disease pathology.

The dysfunction of tolerogenic B cell checkpoints is seen in many autoimmune diseases (87, 88). As has been discussed, these checkpoints, which play a crucial role in restricting the self–reactive potential of the B cell repertoire, have a significant metabolic component (19, 54). It has therefore been investigated whether metabolic dysregulation could compromise the function of these checkpoints. One apparent mechanism underlying defective tolerogenesis is increased exposure to BAFF. Serum BAFF levels are elevated compared to health in several autoimmune diseases, including RA, IgA nephropathy and SLE (89–91). In otherwise healthy mice, autoimmune manifestations are seen following transgenic BAFF overexpression (92). Exposure to excess BAFF rescues self–reactive B cells from deletional checkpoints and supports the survival of anergic B cells (21, 93). Anergic B cells show an increased reliance on BAFF signalling and fail to compete with non–anergic B cells for the cytokine under normal conditions (94). Elevated BAFF may promote their survival, increasing the likelihood of peripheral activation. Regarding metabolism, chronic exposure to BAFF increases the glycolytic and oxidative metabolism of B cells (20). Prolonged exposure to high levels of BAFF may allow self–reactive B cells to avoid the metabolic restriction and energy crisis which contribute to anergy and deletion, respectively.

The autoimmune manifestations of TRAF3–deficient mice further evidence the importance of BAFF in autoimmunity. TRAF3 inhibits BAFF–induced activation of nuclear factor (NF)–κB2 signalling (95, 96). In mice, B cell–specific loss of TRAF3 enhances the survival of resting B cells in a BAFF–independent manner. This results in an expanded B cell compartment, increased spontaneous GC formation and autoimmunity (95). As with exposure to elevated BAFF, TRAF3 deficiency in B cells increases glucose uptake, glycolysis and OXPHOS, in an NF–κB–dependent manner (97).

Another important mechanism underlying B cell anergy is the suppression of PI3K signalling by PTEN, Src homology region 2 domain–containing phosphatase (SHP)–1 and Src homology 2 domain–containing inositol polyphosphate 5–phosphatase (SHIP)–1 (39, 98–100). In mice, PTEN deletion alters the responsiveness of B cells to tolerogenic signals, with activation and proliferation favoured over anergy (39). The loss of either PTEN or SHP–1 in B cells results in autoimmunity (39, 101). Moreover, the expression of PTEN by human B cells is reduced in patients with type 1 diabetes mellitus, autoimmune thyroiditis and SLE compared to healthy controls (98, 102). Together, these results suggest that unrestrained PI3K signalling, through the loss of negative regulators, enables self–reactive B cells to escape tolerogenesis.

Downstream of PI3K, hyperactive B cell mTORC1 signalling is seen in autoimmunity. Given its roles in B cell development, GC reactions and PC function, an association between altered mTORC signalling and autoimmunity is perhaps unsurprising (14, 64). mTORC1 hyperactivity is seen in animal models of autoimmunity, including murine models of SLE and RA (103, 104). In the latter, mTORC1 hyperactivation in B cells was associated with increased glucose metabolism, although disturbed B cell metabolism was deemed to occur downstream of T cell dysregulation (104). In human autoimmunity, elevated mTORC signalling is seen in the B cells of SLE patients, where it is correlated with disease activity, and in the salivary gland B cells of patients with Sjögren’s syndrome (22, 105).

The precise metabolic consequences of dysregulated B cell signalling networks in autoimmunity have not been well defined. However, there is clear evidence to suggest that the tight metabolic control of B cell function is lost in autoimmunity and that this has consequences for pathophysiology. More work is needed to directly investigate the nature and consequences of metabolic dysregulation seen in autoimmune B cells. Furthermore, a more complete characterisation of metabolism in different B cell subsets is needed. Comparatively little is known about the metabolism of B1 cells, B_regs_ and memory B cells in both health and disease. Given the different phenotypes and functions of different B cells, it is likely that they affected differently by metabolic disturbances. Finally, most research has focussed on B cells in SLE, with the dysfunction of B metabolism in other diseases mediated by autoantibodies and B cell cytokines warranting further investigation.

## B Cell Autophagy in Autoimmunity

As discussed above, autophagy promotes metabolic homeostasis of B cells, particularly following peripheral activation and in memory cell maintenance (23, 84). It has also been implicated in antigen presentation and deletional autoimmune checkpoints (75, 81). This has fuelled considerable interest in the role that autophagy may play in expanding the repertoire, functionality and survival of self–reactive B cells in diseases such as SLE, RA, and multiple sclerosis (MS). Although not cell–specific, early evidence of perturbed autophagy in autoimmunity came from genome–wide association studies (GWAS). Specifically, several reports linked single nucleotide polymorphisms (SNPs) in the *Atg5* gene and *Prdm1–Atg5* intergenic region to the development of SLE and, in a European population, to RA (106–109). While GWAS are unable to resolve associations in a cell–specific manner, it was subsequently demonstrated that the B cells of patients with SLE displayed elevated *Atg5* expression compared to healthy controls (110). Furthermore, an SLE–associated SNP in the *Prdm1–Atg5* intergenic region was associated with elevated expression of autophagy–associated genes in the B cells of SLE patients and healthy individuals (110). In parallel to these early GWAS, autophagy was implicated in SLE through therapeutic studies. The 21–mer peptide rigerimod (P140), which ameliorates lupus pathology, was shown to inhibit B cell autophagy (111). Specifically, rigerimod targets chaperone–mediated autophagy, which is upregulated in lupus–prone mice, suggesting that inhibiting dysregulated autophagy may underlie the efficacy of rigerimod (112). The apparent association between B cell autophagy and autoimmunity appears to be mediated by several distinct mechanisms, which may occur in a disease–specific manner.

**Figure 3:**
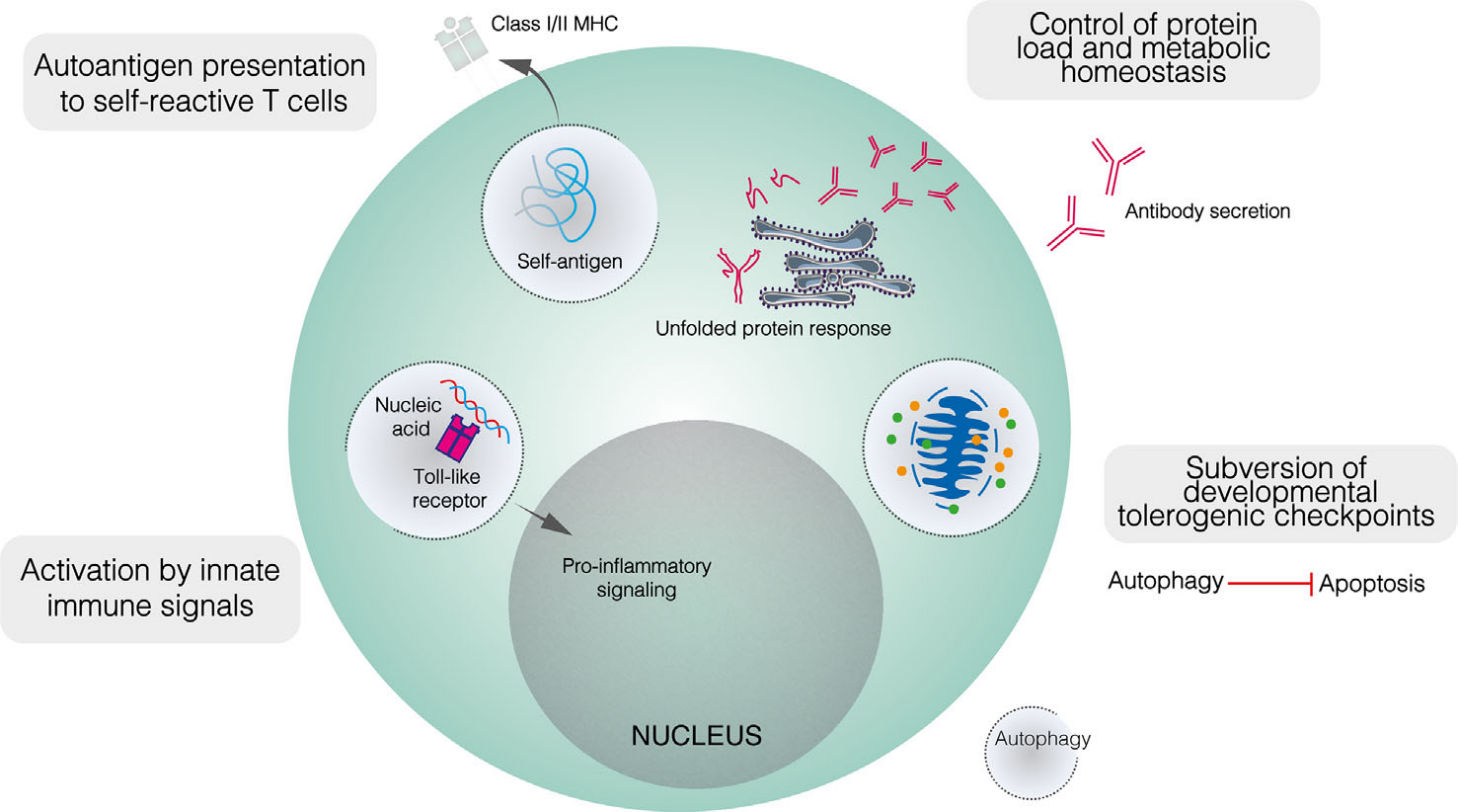
The role of autophagy in the function of self–reactive B cells. Developmentally, upregulated autophagy is thought to enable autoimmune B cell precursors to subvert apoptotic checkpoints. In mature B cells, autophagy appears to mediate both the presentation of self–peptides by B cells, and the recognition of innate immune ligands such as nucleic acids. It is also likely that autophagy contributes to metabolic homeostasis and control of the misfolded protein load in plasma cells.

As with metabolism, dysregulated autophagy has been implicated in the loss of tolerogenic checkpoint function in autoimmunity. While autophagy is dispensable for B2 cell development, this does not preclude a role for altered autophagy in enabling the subversion of metabolic or apoptotic checkpoints by self–reactive B cell precursors (**Figure 3**) (25, 40). Indeed, while autophagy is upregulated in lupus–prone mice compared to healthy controls, this difference is restricted to bone marrow–resident pre–B, immature and mature B cells (25). No difference is seen in among splenic B cells, including PCs. In patients with SLE, elevated autophagy compared to healthy controls is more notable in naïve B cells than PCs or memory cells (25). Both pre–B and naïve B cells are subject to tolerogenic checkpoints. Upregulated autophagy may protect self–reactive B cells against apoptotic or metabolically damaging stimuli. Similarly, the induction of autophagy has been proposed to mediate T cell escape from tolerogenic checkpoints (113).

Autophagy also appears to play a role in enabling B cells to process and present peptides derived from self–antigens to cognate T cells (**Figure 3**). Physiologically, autophagy has been shown to mediate the presentation of peptides derived from particular antigens (81). In autoimmunity, autophagy enables B cells to present citrullinated peptides on MHC–II, antibodies against citrullinated antigens feature prominently in autoimmune diseases, most notably in RA (114, 115). *In vitro*, the presentation of endogenous citrullinated peptides by B lymphoma cells was enabled by serum starvation, a state which activates autophagy, and suppressed by both the class III PI3K inhibitor 3–MA and *Atg5* knockdown (115). In contrast, neither starvation nor 3–MA affected the presentation of non–citrullinated antigens. Furthermore, stimulation of primary B cells with anti–IgM antibodies induced significant citrullinated peptide presentation and increased LC3–II levels (115). Given the frequency of anti–Ig antibodies in RA and the apparent induction of autophagy following BCR engagement, such antibodies have been proposed to trigger the presentation of endogenous citrullinated antigens on MHC–II (115). Although the exact relationship between autophagy and RA remains unclear, B cell autophagosome density is not upregulated in patients with RA compared to healthy controls, yet autophagic activity appears necessary for the presentation of certain autoantigens (116).

B cell autophagy has also been associated with the presentation of citrullinated self–antigens to cytotoxic T lymphocytes in MS. EBV, a well–established risk factor for development of MS, induces autophagy in B cells, and has recently been linked to the class I MHC (MHC–I) presentation of myelin oligodendrocyte glycoprotein (MOG)–derived peptides (24, 117–119). The role of autophagy in this process is poorly defined, although autophagosomes may protect citrullinated MOG peptides from cathepsin–mediated degradation, directing them towards MHC–I cross–presentation machinery (24).

As well as BCR ligands, autophagy has been implicated in B cell recognition of innate immune triggers, specifically nucleic acid antigens, by TLRs 7 and 9. These receptors recognise pathogen–derived ssRNA and CpG–containing dsDNA respectively, with their cellular expression restricted to endosomes to prevent cross–recognition of self–nucleic acids. The recognition of self–nucleic acids may directly activate B cells or sensitise them to BCR–mediated activation: the transgenic overexpression of *Tlr7* or *Tlr9* generates autoreactive PCs and triggers lupus–like disease in mice (26, 120, 121). Murine experimental lupus induced by transgenic *Tlr7* overexpression is ameliorated in the absence of B cell autophagy (122). Autophagy mediates the transport of endocytosed RNA–containing immune complexes to TLR7–containing endosomes in dendritic cells and may play a similar role in B cells (**Figure 3**) (123). Autophagy has also been implicated in TLR9 signalling (124). Following BCR engagement, TLR9–containing endosomes are recruited to autophagosomes which contain the internalised BCR–antigen complex, although this co–localisation is abolished by 3–MA (124). Anti–IgM conjugated to CpG DNA hyperactivates B cells compared to anti–IgM alone, and this additive effect was abolished when TLR9 recruitment to autophagosomes was blocked by disrupting microtubule function (124). Collectively, autophagy seems to play an important role in delivering nucleic acid antigens to endosomal TLRs, potentially impairing the discrimination between self– and non–self–nucleic acids.

Intuitively, the role of autophagy in maintaining PC viability should extend to self–reactive PCs (**Figure 3**) (23, 40, 60). While PC autophagy does not appear to be upregulated in lupus–prone mice compared to healthy controls, it contributes demonstrably to the pathology of murine experimental lupus (25, 40). In mice with *Atg5* conditionally deleted in mature B cells, anti–dsDNA IgM levels were similar to those seen in wildtype mice, whereas anti–dsDNA IgG was significantly lower (40). The splenic B cell repertoire, including the proportions of different B cell subpopulations and the appearance of germinal centres, was largely unaffected by the absence of autophagy, although bone marrow LLPCs were depleted (40). Together, these results suggest that autophagy makes little contribution to early B cell activation in autoimmunity but is important in ensuring the long–term survival of autoreactive PCs. It was proposed that SLPC survival may have been compromised in the absence of autophagy but was compensated for by increased replenishment (40). Importantly, autophagy deficiency likely also reduces the memory B cell compartment (84).

While B2 cell autophagy plays an important role in autoimmunity, its relevance in other B cell subsets remains poorly defined. Although B1 cells display great sensitivity to changes in autophagic flux, it is not clear whether changes in B cell autophagy seen in autoimmunity apply to these cells and what the effects might be (18). This is important to understand, given that self–reactive IgM produced by B1 cells is implicated both in autoimmune pathogenicity and in the clearance of apoptotic cells (8, 11). Moreover, B1 cells in gut–associated lymphoid tissue promote intestinal homeostasis through IgA and IgM production (12). The loss of B cell autophagy reduces mucosal IgA and impairs B cell responses to intestinal inflammation (77). Overall, B1 cell autophagy may be relevant to inflammatory and autoimmune diseases, particularly within the gastrointestinal tract. As in B1 cells, the effects of perturbed autophagy on B_reg_ function have yet to be defined, although their unique phenotype suggests that they may be affected differently to antibody–secreting cells. Further investigation is required to understand the role of autophagy in unconventional B cells during autoimmunity.

## B Cell Metabolism and Autophagy as Therapeutic Targets

The dysregulation of B cell metabolism and autophagy in autoimmune diseases has raised the prospect of targeting these processes therapeutically. While traditional immunosuppressive drugs such as glucocorticoids affect immunometabolism, recent efforts have focussed on disrupting metabolic pathways more specifically (125). Several drugs being explored or approved for the treatment of autoimmune diseases target B cell immunometabolism or its regulators, likely contributing to their efficacy.

As discussed above, elevated BAFF levels are seen in several autoimmune diseases (89–91). B cells exposed to high levels of BAFF have enhanced metabolic capacity and can escape from tolerogenic checkpoints (20, 21). Efforts to inhibit this dysregulated signalling culminated in the approval of the BAFF–specific monoclonal antibody belimumab as an add–on therapy in SLE, having demonstrated efficacy, including ameliorating B cell dysfunction, in phase 3 clinical trials (126). The treatment of SLE patients with belimumab induces anergy in autoreactive B cells (93). Notably, the BAFF–independent survival of memory B cells means that humoral immune responses to vaccine antigens are left intact by belimumab (127). The ability to target self–reactive B cells without compromising physiological B cell responses is clearly desirable. Excessive BAFF signalling may also be attenuated through blockade of its receptor. Recently, the BAFF receptor inhibitor ianalumab was shown to effectively deplete B cells and improve clinical parameters in patients with Sjögren’s syndrome (128).

Hyperactive mTORC1 signalling, a feature of both T and B lymphocytes in autoimmunity, can be inhibited with rapamycin. In murine models of SLE, rapamycin attenuates pathology, including decreasing anti–dsDNA antibody titres (129). Although its effects have been largely attributed to changes in T cells, rapamycin inhibits BAFF–mediated mTORC1 signalling in B cells, limiting proliferation and survival (130, 131). mTORC1 inhibition also results in the induction of autophagy. Although self–reactive B cells appear to benefit from a degree of autophagy upregulation, increased autophagic flux may be detrimental to B cell survival (75). In a single–arm, open–label Phase 1/2 trial in SLE patients, rapamycin was associated with decreased disease activity and reduced levels of some autoantibodies (132).

mTORC dysregulation is also targeted by metformin. Used to treat type 2 diabetes mellitus, metformin activates AMPK and is, therefore, able to downregulate mTORC1 activity. Metformin has shown efficacy in the treatment of lupus–like autoimmunity in mice (133). As well as decreasing T_FH_ and T_H17_ populations, metformin suppressed PC differentiation and GC formation, resulting in lower autoantibody levels (133). In a separate study, metformin given alongside the glycolytic inhibitor 2–DG was shown to ameliorate autoimmune pathology in lupus–prone mice (134). A recent clinical trial into the efficacy of metformin in SLE patients found no efficacy in reducing the incidence of disease flares, although pooled analysis with a previous trial suggested a modest reduction in flare incidence was achieved, warranting further investigation (135, 136).

While some of the therapeutic effects of rapamycin and metformin may derive from targeting B cell autophagy, this process is affected more clearly by rigerimod (111). Rigerimod disrupts chaperone–mediated autophagy, likely affecting the MHC–II–restricted presentation of endogenous antigens to autoreactive T cells (112, 137). In mice, rigerimod suppresses autoimmune responses without compromising anti–viral immunity (138, 139). Although rigerimod showed efficacy in early clinical trials in SLE patients, it failed to meet its primary endpoint in a recent phase 3 clinical trial (140, 141). However, promising therapeutic potential has meant a new phase 3 trial is scheduled to commence in 2021.

Owing to their limited proteasome capacity and high levels of immunoglobulin production, PCs display exquisite sensitivity to proteasome inhibition (60). Proteasome inhibition has been investigated as a potential therapeutic strategy in diseases characterised by autoantibody production. Efforts to target the proteasome have focussed on bortezomib, which was originally developed for the treatment of multiple myeloma. In multiple myeloma, B cells with high immunoglobulin production are disproportionately vulnerable to proteasome inhibition (142). The high immunoglobulin output of self–reactive B cells in autoimmune diseases will likely confer elevated sensitivity to proteasome inhibitors. In a murine model of lupus, bortezomib alleviated autoantibody–driven pathology (143). In small numbers of human participants, bortezomib has shown promise in treating autoantibody–driven diseases, such as anti–NMDA receptor encephalitis and SLE (144, 145).

Although the metabolism and autophagy of self–reactive B cells represent promising therapeutic avenues, there are several challenges associated with such targets. Firstly, a full understanding of the metabolic regulation of B cell function is lacking in both health and disease. Moreover, much of our current knowledge comes from studying B cell metabolism in mice, not humans. A critical goal in the field is uncovering cell–specific metabolic pathways which may be targeted more selectively. Similarly, immune cells differ considerably in their metabolic plasticity and therefore resistance to metabolic pathway inhibition. However, many of the most commonly used drugs in medicine (e.g. metformin, statins, and methotrexate) all target broadly active metabolic processes. This observation suggests that tolerability of metabolic inhibition be better than supposed. As always when treating autoimmunity, excessive immunosuppression must be avoided, and the risk of infection will remain an important consideration. Finally, the extent to which specific metabolic pathways are disturbed in patients with autoimmune disease is likely to vary significantly between individuals. The use of metabolomic biomarkers may be needed to identify patients who are likely to respond to treatments targeting metabolism.

## Conclusion

Although metabolism has emerged relatively recently as a regulator of immunological function, it is clear that it has far–reaching consequences in both the physiological state and autoimmunity. Arguably, efforts have been made to exploit B cell metabolism and autophagy therapeutically before these processes have been well characterised. Nevertheless, it appears that they do offer real translational potential. While much focus to date has been rightly placed on understanding the role of metabolic regulators such as mTORC1 and AMPK, metabolism itself, as well as its impact on B cell function, require greater investigation. Finally, it is imperative that metabolic interactions between B cells and other leukocyte populations are explored. While this review has focussed only on B cells, exploiting the full therapeutic potential of B cell metabolism in autoimmune diseases will require it to be understood within the context of wider immune dysfunction.

## Author Contributions

IR wrote and edited the manuscript AC supervised and edited the manuscript. All authors contributed to the article and approved the submitted version.

## Funding

AC is supported by a Wellcome Trust award (211072/Z/18/Z).

## Conflict of Interest

The authors declare that the research was conducted in the absence of any commercial or financial relationships that could be construed as a potential conflict of interest.
